# Research progress in predicting the conversion from mild cognitive impairment to Alzheimer’s disease via multimodal MRI and artificial intelligence

**DOI:** 10.3389/fneur.2025.1596632

**Published:** 2025-06-02

**Authors:** Min Ai, Yu Liu, Dan Liu, Chengxi Yan, Xia Wang, Xun Chen

**Affiliations:** ^1^Department of Anesthesiology, Nanan District People’s Hospital of Chongqing, Chongqing, China; ^2^Department of Radiology, Chongqing Public Health Medical Center, Chongqing, China; ^3^Department of Cardiology, The First Affiliated Hospital of Chongqing University of Chinese Medicine, Chongqing, China; ^4^Department of Radiology, The Second Affiliated Hospital of Xi'an Jiaotong University, Xi’an, Shaanxi, China; ^5^Department of Radiology, Chongqing Western Hospital, Chongqing, China

**Keywords:** mild cognitive impairment, Alzheimer’s disease, multimodal MRI, artificial intelligence, conversion

## Abstract

Predicting the transition from mild cognitive impairment (MCI) to Alzheimer’s disease (AD) has important clinical significance for dementia prevention and improving patient prognosis. Multimodal magnetic resonance imaging (MRI) techniques (including structural MRI, functional MRI, and cerebral perfusion MRI) can yield information on the morphology, structure, and function of the brain from multiple dimensions, providing a key basis for revealing the pathophysiological mechanisms during the conversion from MCI to AD. Artificial intelligence (AI) methods based on deep learning and machine learning, with their powerful data processing and pattern recognition capabilities, have shown great potential in mining the features of multimodal MRI data and constructing prediction models for MCI conversion. Therefore, this paper systematically reviews the research progress of multimodal MRI techniques in capturing brain changes related to MCI conversion, as well as the practical experience of AI algorithms in constructing efficient prediction models, analyses the current technical challenges faced by the research, and discusses future directions, with the goal of providing a scientific reference for the early and accurate prediction of MCI conversion and the formulation of intervention strategies.

## 1 Introduction

**Figure 1:**
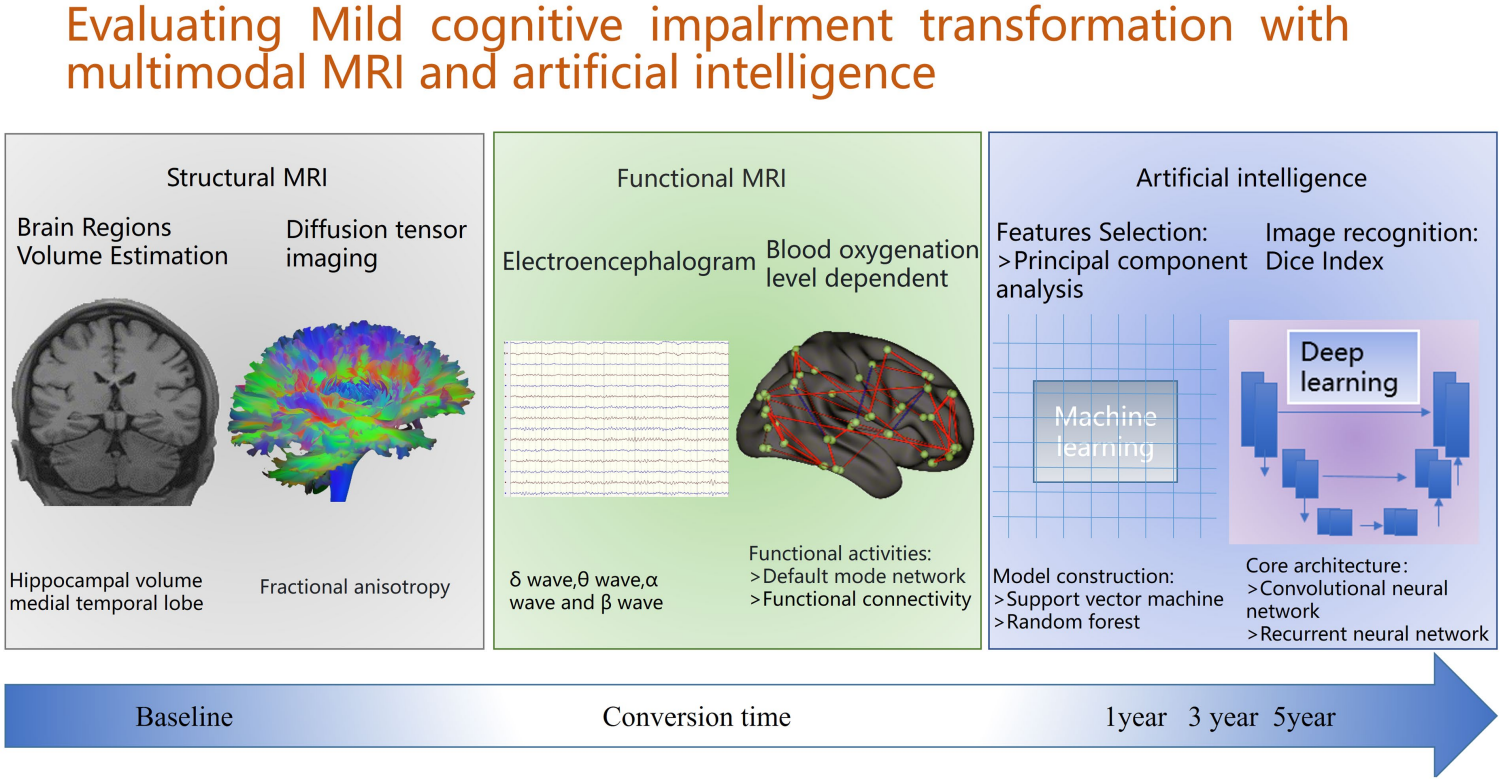
Overview of predicting conversion from mild cognitive impairment to Alzheimer’s disease: including structure MRI, functional MRI and artificial intelligence.

According to the World Health Organization, approximately 50 million people worldwide suffer from dementia, and it is projected that by 2050, the number of people with dementia will reach 152 million (1). The main characteristic of dementia is the progressive deterioration in multiple cognitive domains, which is severe enough to interfere with daily functions (2). Alzheimer’s disease (AD) is one of the most common causes of dementia, accounting for approximately 75% of the total number of dementia cases (3–5). The symptoms of AD start with mild memory difficulties and evolve into cognitive impairment, impairment in complex daily activities, and deficits in several other aspects of cognition (6). When AD is clinically diagnosed, neuronal loss and neuropathological lesions have already occurred in many areas of the brain (7). Therefore, the key to delaying potential damage is to intervene in a timely manner before AD progresses to mild symptoms, delaying the onset of irreversible dementia. MCI leads to cognitive impairment at an intermediate stage, between those with normal memory changes associated with ageing and those with obvious AD (8). At this very early stage, individuals still have sufficiently intact cognitive functions that can be utilized and guided to compensate for or restore functions (9). Although MCI is currently considered an early stage of AD and does not affect the normal life of patients, approximately 10–20% of patients convert to AD each year (10). If progressive MCI patients can be identified at an early stage, providing a “window of opportunity” for the prevention and treatment of AD, it may be possible to prevent its conversion to AD, which will be highly important for the treatment and prognosis of AD (11). Many previous in depth studies have shown that multimodal MRI techniques (12), including structural MRI (13), functional MRI, and cerebral perfusion MRI, each have unique advantages (14–16). They can comprehensively and meticulously acquire abundant information on the morphology, structural function and cerebral blood flow of the brain from multiple dimensions and directions. The information obtained by these multimodal MR images complements each other, providing a theoretical basis for deeply revealing the pathophysiological changes in the cells, tissues, and functions of the brains of MCI patients and offering new perspectives for further exploration of the pathogenesis of MCI patient transformation and subsequent clinical diagnosis and treatment strategies. In addition, AI technologies, such as deep learning and machine learning (17–19), are pioneering a revolutionary wave in the medical field due to their unparalleled powerful capabilities (20, 21). Deep learning relies on a deep neural network architecture and can automatically mine deep-level features from massive and complex data without the need for manual predecision of feature extraction rules, resulting in a high degree of adaptability and flexibility (22). Machine learning (23), through various algorithms, involves the learning and training of data to achieve tasks such as classification and prediction of unknown data (24). Many studies have shown that prediction models constructed based on AI have great potential in predicting the risk and time nodes of MCI conversion to AD, providing strong technical support for early intervention in MCI and delaying the conversion of AD, and are expected to promote breakthrough progress in precision medicine in this field. Multimodal MRI techniques, including structural, functional, and cerebral perfusion MRI, can yield brain information from multiple dimensions, providing a theoretical basis for revealing pathological changes in MCI patients (25). AI techniques such as deep learning and machine learning are revolutionary in the medical field (26). Prediction models constructed based on AI have great potential in predicting the conversion of MCI to Alzheimer’s disease (AD), which can provide technical support for early intervention and promote the development of precision medicine. Therefore, this paper comprehensively and deeply summarizes the latest research progress on brain changes related to the prediction of MCI to AD conversion through multimodal integration plus AI transformation. It is hoped that this information will assist clinicians and researchers in gaining a deeper understanding of the underlying mechanisms of MCI conversion to AD, thereby facilitating the development of more effective early intervention measures and providing new hope for improving the quality of life and prognosis of MCI patients (Figure 1).

## 2 Research progress in the use of brain structure MRI for the prediction of MCI conversion

Clinically, structural magnetic resonance imaging (sMRI) is widely used in the diagnosis and prediction of MCI (27). sMRI, a crucial component of multimodal magnetic resonance imaging techniques, primarily encompasses two core aspects: image-based brain morphometric measurements and structural data integration. The methods of image-based brain morphometric measurements are diverse and include voxel-based, surface-based, cortical folding, and white matter microscopy measurements. Numerous studies have shown that the volume changes derived from voxels in specific regions of the brain are closely related to the conversion process of MCI to AD. In a study conducted by Tapiola et al., MCI patients with a 34-month follow-up were selected to explore the predictive value of different methods for the conversion of MCI to AD (28). During this research, multiple indicators, such as MRI-derived medial temporal lobe (MTL) structure volumes, white matter (WM) lesions, Mini-Mental State Examination (MMSE) scores, and APOE genotypes, were comprehensively analysed. The final findings indicated that only the MTL volume could effectively predict high-risk patients for MCI conversion to AD. Similarly, the study by deToledo-Morrell et al. (29) focused on this area. In this study, 27 MCI patients were followed up for 36 months after baseline diagnosis, and 10 of them progressed to AD during this period. Researchers have compared the volumes of the hippocampus and entorhinal cortex to determine which of these two regions is more advantageous in differentiating between stable and progressive MCI patients. The results showed that both the hippocampal and entorhinal cortex volumes had predictive capabilities, but the entorhinal cortex volume performed better in prediction, with a prediction accuracy as high as 93.5%. Costafreda SG et al. (30) developed an application tool that can automatically extract the 3D hippocampal shape morphology for predicting the conversion of MCI to AD. The model they constructed performed well in predicting the conversion of MCI patients to dementia within 1 year, with an accuracy rate reaching 80%. In addition, changes in cortical thickness can also be used to predict the conversion of MCI to AD. Bakkour A reported that thinning of the temporal and parietal lobes could predict the conversion of MCI to AD, with a sensitivity of 83% and a specificity of 65% (31). Desikan RS (32) reported that automatic MRI measurements of the medial temporal lobe cortex could accurately and reliably predict the conversion time of MCI. As a predictor of clinical decline, it is superior to cellular and metabolic measurements and is expected to become a predictive biomarker for AD. Notably, microscopic changes in white matter cannot be observed by the naked eye. Texturometry is a relatively abstract concept; it describes the spatial and statistical relationships of pixel values in the region of interest (ROI), which can quantify features such as smoothness, roughness, and regularity, thereby reflecting subtle structural changes in white matter. In the early stages of AD, subtle changes in neurofibrillary tangle (NFT) and amyloid-*β* (Aβ) plaque deposition occur. These minor changes can form specific texture patterns in MR images. By using the technical means of extracting texture descriptors from the images, these texture patterns can be identified, providing important clues for the early diagnosis of AD. Tang et al. (33) integrated the structural information of the whole brain, used FreeSurfer software to extract parameters such as the cortical surface area, average thickness, folding index, and grey matter volume of each subregion of the whole brain, and further extracted the radiomic parameters of the subcortical brain regions. On this basis, they developed a radiomic-clinical-laboratory model that can accurately predict whether MCI will progress to AD and the time of conversion, with a C-index as high as 0.07. This achievement is highly important for formulating personalized treatment plans and delaying the occurrence of irreversible dementia. Shu ZY et al. (34) also developed a comprehensive model based on whole-brain (white matter, grey matter, cerebrospinal fluid) radiomics, which can accurately identify and predict high-risk groups of MCI patients who may progress to AD. However, the integration of brain structural data is also commonly used for predicting the conversion of MCI. Two main methods are used: diffusion tensor imaging (DTI) and neurite orientation dispersion and density imaging (NODDI). Song Q et al. (35) utilized DTI technology to construct a brain network based on white matter fibre tracts and extract network attribute features from it. They subsequently downscaled these white matter network features, used a comprehensive downscaling method to construct white matter markers, and then combined clinical features and performance evaluations to develop a comprehensive model. This comprehensive model has demonstrated excellent performance, with diagnostic efficacies of 0.924 and 0.919 in the training group and the experimental group, respectively. This model can effectively identify high-risk patients with MCI Conversion to AD, provides valuable auxiliary biomarkers for the early detection of AD, and is highly important in the field of early AD diagnosis. The theoretical basis of traditional DTI assumes that the diffusion of water molecules in all directions is free and unrestricted, i.e., Gaussian motion (36). However, in actual human tissues, the diffusion of water molecules is not completely free or unrestricted. Cell membranes and organelles restrict the free diffusion of water molecules, i.e., non-Gaussian motion. Therefore, two important DTI parameters, fractional anisotropy (FA) and mean diffusivity (MD), both lack specificity (37). In recent years, the emerging NODDI technique has been used to observe the microstructure of brain tissue more sensitively and specifically, showing superior performance in detecting grey matter and white matter lesions (38). This technique can distinguish three microstructural tissue models: intracellular water, extracellular water, and cerebrospinal fluid (39). The diffusion of water in neurites is restricted, the diffusion of water outside neurites is hindered, and the diffusion of water molecules in cerebrospinal fluid is completely free. The diffusion of water molecules in each compartment does not affect each other, so independent standardized MR parameters, such as the neurite density index (NDI), orientation dispersion index (ODI), isotropic volume fraction (Viso), and extracellular volume fraction (Vec), can be obtained. As a neurodegenerative disease, the main pathological changes associated with AD are the degeneration and loss of neurons and neurites, which irreversibly worsen over time. Until the middle and late stages, many neurons are lost, causing gross atrophy of the brain parenchyma. Some researchers have reported that NODDI indicators may be sensitive markers of pathological changes in early-stage AD patients (40, 41). Nicholas et al. used the NODDI technique to explore the microstructure of the brain parenchyma in MCI and AD patients and reported that the NDI in the temporal and parietal cortical regions of MCI patients was significantly decreased, whereas in AD patients, the NDI and ODI in the parietal, temporal, and frontal cortical regions were significantly decreased. After controlling for cortical thickness, when the microstructure in the same brain region was compared, differences in NODDI indicators were also found between MCI and AD patients. In addition, for MCI patients with normal cortical thickness, the cortical NDI in the temporal, parietal, and posterior cingulate regions was also decreased (42). The NODDI indicators can detect not only macroscopic structural changes but also pathological changes in the microstructure of the cortex in MCI patients and early-stage AD patients before macroscopic structural changes occur. With respect to the exploration of white matter regions, Fu et al. used NODDI and DTI diffusion models to explore microstructural changes in the white matter regions of normal, MCI, and AD individuals. Compared with those in normal people, the NDI and ODI values in the MCI and AD groups were significantly lower, and the Viso value was significantly greater. However, there was no significant difference in the FA value in the MCI group, whereas the FA value in the AD group was significantly lower than that in the normal group. NDI and ODI are more sensitive to white matter microstructural changes than FA (41). Therefore, we believe that the NODDI technique, which is based on sMRI, can quantify the complexity of the microstructures of neurites and axons in MCI patients, thereby providing morphological information on nerve fibres. It has increased specificity and sensitivity in the evaluation of the microstructure of brain tissue, providing a basic guarantee for studying the risk of MCI patients transitioning into AD patients.

## 3 Research progress on functional magnetic resonance imaging-assisted MCI transformation prediction

Functional brain imaging techniques are important tools for exploring the mysteries of the brain and revealing the laws of neural activity, playing a crucial role in the process of MCI conversion. Functional brain imaging devices can generally be divided into two categories, one of which constitutes devices that measure the electromagnetic fields generated by neuronal activities. These devices can achieve real-time monitoring and localization of brain neural activities by capturing the weak electromagnetic field signals generated by neuronal electrical activities. Among them, electroencephalograms (EEGs) and magnetoencephalograms (MEGs) are the most representative devices and have been widely used to study changes in the brain functions and connections of MCI patients. Mazaheri A et al. (43) reported that subtle abnormalities in the EEG activities of MCI patients during a word comprehension task can serve as evidence for the conversion of MCI to AD. Subtle malfunctions in the brain network that support language comprehension may indicate the conversion of MCI. Poil SS et al. (44) reported that multiple EEG biomarkers associated mainly with activities in the *β*-frequency range (13–30 Hz) could predict the conversion from MCI to AD. They also proposed combining multiple EEG-based neuromarkers into a diagnostic classification index, which could better predict the conversion from MCI to AD. Yu M et al. reported that the MEG-based resting-state multiplex networks in AD patients were preferentially disrupted in hub regions, including the medial temporal lobe (left hippocampus), posterior default mode network, and occipital regions. Pusil S et al. (45) employed MEG phase-based multivariate coupling measures to independently construct dynamic functional connectivity networks in five classical frequency bands. Thus, the distance between the fluctuations in functional strength for each pair of regions of interest (ROIs) in the two conditions was calculated through dynamic time warping (DTW), which extracts many features. Machine learning algorithms were used to reveal 30 DTW-based features in the five frequency bands, which could be used to predict the conversion from MCI to AD. We argue that strategies to increase prediction efficacy are shifting from single-modality optimization to cross-modal integration. By combining the temporal resolution advantages of EEG with the spatial localization precision of MEG and constructing a multimodal recognition system through well-designed fusion algorithms, it is possible to achieve synergistic integration of complementary information from both modalities. This innovative approach not only has the potential to exceed the performance limits of single modalities but also opens new research paradigms for MCI conversion prediction, holding significant exploratory value and application prospects in the future development of neuroimaging technologies.

The other category consists of devices sensitive to the haemodynamic or metabolic effects of neuronal activities, which indirectly reflect the functional state of the brain based on the coupling relationship between neuronal activity and local cerebral blood flow or blood oxygen metabolism. Functional magnetic resonance imaging (fMRI) and positron emission tomography (PET) are typical examples of such devices. fMRI is based on the blood oxygenation level-dependent (BOLD) signal to measure the haemodynamic changes generated during brain activity, thus indirectly representing the activities of brain neurons (46). BOLD-fMRI includes resting-state functional magnetic resonance imaging (rs-fMRI) and task-based fMRI (47, 48). Common brain regions with abnormal brain function in MCI patients include the precuneus, posterior cingulate cortex, lingual gyrus, parahippocampal gyrus, and temporal lobe (49, 50). The precuneus, posterior cingulate cortex, and inferior parietal lobule are key brain regions for Aβ deposition in MCI patients and are closely related to cognitive decline and disease conversion (51). Pasquini L et al. divided the default mode network (DMN) into the anterior DMN and the posterior DMN and reported that the functional connectivity (FC) of the posterior DMN was positively correlated with the level of Aβ deposition in the whole-brain cortex; specifically, the higher the FC of the posterior DMN is, the greater the degree of Aβ deposition in the brain region. With disease conversion, Aβ deposition peaks in the early stage of MCI, indicating that Aβ is more likely to be deposited in brain regions with high functional connectivity (52). This discovery provides insight into the early deposition of Aβ in the DMN, suggesting that rs-fMRI is helpful for understanding the pathophysiological mechanism of AD and can be used as a powerful tool to identify different stages of AD. S Kemik K (53) investigated functional changes associated with MCI via independent component analysis (ICA), word-generation task fMRI, and resting-state fMRI. The results showed that in the resting-state fMRI data, the language network exhibited larger voxel sizes in the bilateral lingual gyri than did the task-based word-generation fMRI data. In task-based fMRI, the right temporo–occipital fusiform cortex, right hippocampus, and right thalamus were also activated. Reduced activation in the dorsal attention network (DAN) and visual network was observed in MCI patients during word-generation task-fMRI. Therefore, task-based fMRI combined with ICA serves as a more sophisticated and reliable tool for evaluating cognitive impairments in language processing. Therefore, in our view, fMRI is one of the most widely used neuroimaging techniques for studying abnormal brain function activities in MCI and AD patients. fMRI has a significant correlation with pathological biomarkers and can reflect the pathological characteristics of the brain, providing the possibility of finding noninvasive markers for the transformation of MCI patients to AD patients. PET is also a crucial functional brain imaging device. Metabolic activity in different brain regions can be detected by injecting radiolabelled tracers into the body. PET can reflect the physiological and pathological processes of the brain at the molecular level, offering unique advantages in predicting the conversion to MCI. When patients present with clinical dementia symptoms, reductions in the cerebral glucose metabolism rate (MRglc), which are easily detectable via 2-[^18^F]fluoro-2-deoxy-D-glucose positron emission tomography (FDG-PET) examinations, have already occurred in the association cortex. Abnormal phosphorylation of the tau protein is an important mechanism for the progressive worsening of MCI. Arnáiz E (54) suggested that reductions in temporoparietal regional reduced glucose metabolism (rCMRGlu) and measurements of visuospatial function may help predict the evolution of MCI patients to AD patients. Groot C (55) reported that tau PET performed best as an independent biomarker for predicting dementia conversion in MCI patients and that tau PET scans may currently be the best available neuroimaging biomarker. Souchet B demonstrated that the b-healing test using multiomics blood biomarkers exhibits high predictive specificity in identifying AD patients within cognitively impaired populations, maximizing the reduction in false positives. When used in conjunction with amyloid screening, it can effectively identify an almost pure cohort of MCI individuals (56).

Brain functional imaging techniques play crucial roles in monitoring the conversion of MCI. However, current single-modality technologies have inherent limitations, such as ambiguity in the spatial localization of EEGs and task-dependent fMRI. In this context, multimodal fusion has emerged as a transformative approach. By integrating the complementary strengths of different modalities and leveraging machine learning to synthesize cross-modal features, more accurate predictive models can be constructed. These models provide fundamental support for early intervention and personalized prediction in MCI, driving advancements in both research and clinical applications.

## 4 Cerebral haemodynamic changes during transformation in patients with MCI

Numerous studies have confirmed that cerebrovascular dysfunction is an important mechanism in the occurrence and development of MCI and AD and that changes in cerebral perfusion are closely related to the conversion of MCI (57, 58). Arterial spin labelling (ASL) is a technique based on the theoretical hypothesis that contrast agents can freely diffuse from blood vessels into tissue spaces (59); it uses the difference in signal intensity generated before and after magnetically labelled arterial blood water protons flow into the imaging plane to obtain cerebral MRI perfusion images (60). Currently, it has become a clinically recognized imaging examination method for MCI and AD patients (61). An increasing number of studies have shown that cerebral blood flow is closely related to the course of AD and MCI, pathological markers, vascular pathological burden, the apolipoprotein E (ApoE) ε4 allele, and the conversion of cognitive impairment (62). ASL-based studies have shown that, compared with healthy controls, MCI patients have increased blood flow in the bilateral hippocampus, precuneus, and left middle temporal gyrus (63) and increased blood flow in the right hippocampus and temporoparietal cortex (64). As the disease progresses to the stages of mild cognitive impairment and dementia, blood flow in the posterior cingulate gyrus, precuneus, and temporoparietal cortex decreases, and the hypoperfusion area subsequently extends to the frontal and occipital lobes (65). Therefore, changes in cerebral blood flow in MCI patients can simultaneously reflect neurodegeneration and vascular pathological burden. The change in cerebral blood flow during the conversion of MCI to AD shows an inverted “U” shaped curve; that is, cerebral blood flow compensatorily increases in the early stage of the disease and then gradually decreases. The ASL-based examination technique can reflect the process of MCI transformation to AD by measuring cerebral blood flow. In addition, there are also bottlenecks in the ASL technique; specifically, the ASL sequence is sensitive to motion, and its post processing requires complex haemodynamic modelling, which hinders its clinical application. Therefore, it is necessary to combine it with biomarkers such as amyloid PET to increase its specificity.

Therefore, the use of multimodal MRI technology is a reliable method for detecting the brain structural, functional, and cerebral blood flow characteristics of patients with MCI transforming to AD. This method provides a powerful guarantee for the risk assessment of whether and when MCI patients will transform to AD and offers a more scientific basis for further exploration of the pathophysiological mechanism, early diagnosis, treatment, and prognosis of MCI transforming to AD.

## 5 Deep learning and machine learning AI methods build a new framework for transforming MCI risk prediction into AD risk prediction

Deep learning is an ideal tool for big data processing. It can efficiently handle massive amounts of data, mine valuable information therein, and elucidate the relationships within the data (66). Therefore, in the face of complex, high-dimensional, and heterogeneous biomedical data and diverse clinical tasks, deep learning has significant advantages and broad application prospects. Deep learning is a rapidly emerging research tool in the biomedical sciences and is currently widely used to predict the conversion of MCI to AD. Yue L et al. (67) employed deep learning techniques to explore subtle structural changes in the brains of patients with MCI in depth. They also constructed a unique deep learning framework based on sMRI to predict the disease conversion of MCI. This framework demonstrated excellent performance, with an AUC as high as 0.812. By combining deep learning algorithms with MR images, this study successfully achieved effective prediction of the conversion from a normal cognitive state to MCI in individuals. Deep learning methods have great potential for exploring key brain changes in the early stage of MCI; this not only helps to analyse the occurrence and development mechanism of the disease more deeply but also opens new directions and ideas for subsequent related research, which is promising for promoting more in-depth research studies in this field. Machine learning (ML) is the scientific study of algorithms and statistical models used by computer systems to perform specific tasks effectively without using explicit instructions, relying instead on patterns and inferences (24, 68). ML is regarded as a subset of AI. A mathematical model constructed by a machine learning algorithm based on sample data, known as “training data,” is used to make predictions or decisions without being explicitly programmed to perform the task. Commonly used machine learning algorithms include logistic regression, K-nearest neighbours, random forest (RF), support vector machine (SVM), and decision tree (DT) (69). Currently, machine learning algorithms for the study of MCI diagnosis, differential diagnosis, and transformation risk assessment show great potential (70, 71). Feng et al. also reported that hippocampal radiomic biomarkers and the established radiomic model are highly important for the diagnosis, differential diagnosis, and treatment of MCI patients (72). Barnes DE developed a Cox model based on cortical thickness, the hippocampus, and neurological scales to predict the likelihood of transformation from MCI to AD (73). Skolariki K et al. (74) retrospectively analysed 803 participants from the ADNI cohort. Three different ML models, namely, the SVM, decision tree (DT), and naive Bayes (NB) models, were employed to predict the conversion of MCI, achieving a prediction accuracy rate of 84%. Moradi E et al. (75) used a semisupervised learning method to develop a novel MRI biomarker for predicting the conversion from MCI to AD. Subsequently, with the help of supervised learning algorithms, they integrated this biomarker with the subjects’ age and cognitive measurement data to generate an aggregated biomarker. Finally, they constructed a prediction model using the RF algorithm to predict the conversion from MCI to AD, and the accuracy of this model was as high as 0.90. AI technologies, with their powerful data processing capabilities, can handle vast amounts of complex data and uncover hidden patterns and relationships within them, thereby increasing the accuracy of predicting the conversion of MCI to AD. Compared with traditional clinical prediction methods, AI-based prediction models can take into account a wider range of factors, including multimodal data (such as clinical, imaging, and biomarker data), reducing the interference of human factors and providing more objective and accurate prediction results. By analysing the longitudinal data of MCI patients, AI models can capture subtle trends in early-stage disease changes, enabling early warning of the conversion from MCI to AD, which is conducive to improving patient outcomes.

However, the artificial intelligence models currently available still have the problem of interpretability of their output results. Doctors require training in interpreting AI output results, for example, in how to make comprehensive judgements by integrating imaging features and clinical scale scores. Moreover, the AI platform interfaces of different medical institutions are inconsistent. Therefore, data input/output specifications (such as DICOM-format annotation) need to be formulated to prevent misjudgment caused by data-format discrepancies. Since the definition of legal responsibility for AI prediction results is unclear, clinical application guidelines and quality control systems must be established.

In addition, most of the current AI models that combine deep learning and machine learning focus on changes in brain structure while ignoring changes in key indicators such as brain function and cerebral blood flow. Therefore, we believe that if deep learning and machine learning techniques can be combined to comprehensively analyse relevant indicators related to the transformation of MCI to AD, such as the whole-brain structure, brain function, and cerebral blood flow, and a model for predicting when MCI patients will transform to AD can be established, it will greatly promote the personalized diagnosis and treatment of MCI patients in clinical practice, which has important clinical significance.

## 6 Challenges faced by AI technology

### 6.1 Data quality and standardization

AI technology is highly dependent on the quality of imaging data. There are differences in the models of imaging equipment, scanning parameters, and image reconstruction algorithms among different medical institutions, which may lead to uneven image quality. Variances in magnetic field intensities (1.5 T/3.0 T), gradient systems, and coil types can lead to discrepancies in image contrast and resolution. For example, hippocampal volume measurements in structural MRI show a coefficient of variation of 12–15% across different devices. Differences in the AIF models used in various studies may introduce biases in the results. Head motion artifacts are common in multimodal scanning, especially during long duration fMRI scans. Their impact should be minimized through standardized head support fixation and motion correction algorithms. Moreover, the standards for image segmentation are not unified. There are differences in manual segmentation by different physicians or in semiautomatic and fully automatic segmentation, which also affects the results. To solve the above problems, it is necessary to establish a unified scanning system and image segmentation standard, carry out prospective multicentre collaborative research, and improve the stability and generalizability of artificial models through large sample analysis.

### 6.2 Feature selection and model optimization

Currently, there are numerous index parameters in deep learning and machine learning, and how to screen out the key indicators remains a considerable challenge. Different studies use different parameter selection methods and model construction algorithms, resulting in poor generalization of research results. In addition, many machine learning models are prone to overfitting, especially when the sample size is small. Therefore, it is necessary to explore more efficient parameter selection algorithms and model optimization strategies to improve the prediction performance and stability of the model. Moreover, it is necessary to vigorously strengthen the external verification of the model with multicentre large samples to ensure the reliability of the model in diverse populations and complex clinical environments.

### 6.3 Clinical translation and application

In the exploration of methods for predicting the conversion of MCI, AI has emerged as a core technology to overcome the bottlenecks of traditional diagnosis. Brain multimodal imaging analysis represents a significant breakthrough in the early diagnosis of AD. AI technology has achieved a leap from “naked-eye recognition” to “intelligent analysis” (44, 55). In the clinical practice of predicting the conversion of MCI to AD, AI models based on deep learning and machine learning demonstrate remarkable potential for clinical transformation due to their ability to mine multimodal imaging data in detail. Studies have shown that structural changes such as hippocampal atrophy and thinning of the entorhinal cortex are important markers of the conversion of MCI (76, 77). Through deep learning models and machine learning algorithms, pixel-level feature extraction can be performed on brain MR structural images, precisely capturing these subtle changes and establishing AI prediction models (78, 79). Moreover, AI can continuously track patients’ longitudinal cognitive test data, automatically focusing on the key time points and feature combinations related to the decline in cognitive function, and dynamically elucidate the potential patterns underlying the conversion of MCI to AD. These achievements not only reveal the potential laws governing the conversion and conversion of MCI but also provide reliable risk assessment tools for clinicians. Although deep learning and machine learning have made some progress in predicting MCI transformation in research settings, there are still many challenges, from research theory to clinical practice. Currently, most studies use a retrospective analysis method, with a limited sample size and a serious lack of prospective, multicentre large-scale clinical verification. Moreover, the practicality and operability of AI models in clinical scenarios still need to be further evaluated. How to integrate research results deeply into clinical work and provide clear, easy-to-understand, and easy-to-use decision-making bases for clinicians has become the core focus for achieving clinical translation. The development of cross-modal transfer learning models and the integration of clinical data with imaging biomarkers can help doctors better understand the current AI scenarios. In addition, it is necessary to properly solve the cost effectiveness problem of related technologies to ensure the feasibility and sustainability of AI technology in the process of clinical promotion.

## 7 Conclusion

In conclusion, multimodal MRI technology has opened a new direction for the noninvasive prediction of MCI conversion and provided support for predicting the transition from MCI to AD. The advancement of AI technology, especially the optimization of deep learning algorithms, will enhance the ability of image analysis and model performance. In addition, strengthening multicentre collaboration and establishing a large-scale standardized MCI database can lay a solid foundation for the clinical translation of AI. The deep integration of AI and multimodal MRI technology may play a crucial role in the clinical diagnosis and treatment of MCI conversion prediction, helping to improve the prognosis of patients, enhance their quality of life, and promote the development of early interventions and treatments for AD.
